# Septic Arthritis of the Temporomandibular Joint (SATMJ) in Adults: A Systematic Review of Case Reports and Case Series, Part I: Etiology and Epidemiology

**DOI:** 10.3390/jcm15020706

**Published:** 2026-01-15

**Authors:** Karolina Lubecka, Kacper Galant, Maciej Chęciński, Kamila Chęcińska, Filip Bliźniak, Agata Ciosek, Tomasz Gładysz, Katarzyna Cholewa-Kowalska, Dariusz Chlubek, Maciej Sikora

**Affiliations:** 1 Department of Oral Surgery, Preventive Medicine Center, Komorowskiego 12, 30-106 Krakow, Poland; lubeckarolina@gmail.com (K.L.); fblizniak@gmail.com (F.B.); 2 Faculty of Medicine, Medical University of Lodz, Al. Kościuszki 4, 90-419 Lodz, Poland; kacpergalant.ld@gmail.com (K.G.); agata.ciosek@stud.umed.lodz.pl (A.C.); 3 National Medical Institute of the Ministry of the Interior and Administration, Wołoska 137, 02-507 Warsaw, Poland; maciej.checinski@pimmswia.gov.pl (M.C.); kamila.checinska@pimmswia.gov.pl (K.C.); sikora-maciej@wp.pl (M.S.); 4 Department of Maxillofacial Surgery, Hospital of the Ministry of the Interior and Administration, Wojska Polskiego 51, 25-375 Kielce, Poland; 5 Department of Oral Surgery, Medical College, Jagiellonian University, Montelupich 4, 31-155 Krakow, Poland; t.gladysz@uj.edu.pl; 6 Department of Glass Technology and Amorphous Coatings, Faculty of Materials Science and Ceramics, AGH University of Krakow, Mickiewicza 30, 30-059 Krakow, Poland; cholewa@agh.edu.pl; 7 Department of Biochemistry and Medical Chemistry, Pomeranian Medical University, Powstańców Wielkopolskich 72, 70-111 Szczecin, Poland

**Keywords:** temporomandibular joint, septic arthritis, temporomandibular disorders, bacterial infection, fungal infection

## Abstract

**Background/Objectives:** Septic temporomandibular joint disease (STMJ) is a rare condition with a potentially dangerous course. Its etiology includes bacterial and fungal infections, systemic factors (e.g., diabetes, immunodeficiencies), and molecular mechanisms. **Methods**: Reports of SATMJ in adults, clinically and microbiologically confirmed, published up to the time of protocol registration (PROSPERO CRD42024613462), were included. ACM, BASE, CENTRAL, PubMed, ClinicalTrials.gov, Embase, Scopus, Google Scholar, and reference lists were searched. The search included strategies using the terms “temporomandibular joint septic arthritis” and related phrases. Two independent reviewers studied a selection of articles and extracted data (demographics, microbiology, risk factors, molecular mechanisms). Risk of bias was assessed using JBI tools, and the certainty of evidence was assessed using the GRADE tool. **Results**: The analysis included 59 cases of SATMJ. Anaerobic infections were found in 77%, Gram-positive infections in 72%, and fungal infections in only 7%. Diabetes and immunoincompetence were associated with SATMJ. **Conclusions**: The results highlight the predominance of Gram-positive and anaerobic infections. Systemic factors, such as diabetes, increase the risk of SATMJ. Limitations result from the heterogeneity and retrospective nature of the analyzed cases and possible publication biases.

## 1. Introduction

### 1.1. Background

The temporomandibular joint (TMJ) is a bilateral structure involved in many important processes for the body, including articulation, which enables communication, and chewing, which allows digestion to begin [1,2]. It is distinguished by its unique anatomy as it is the only joint in the human body with two compartments: upper and lower [1,3]. The compartments are separated from each other by the articular disc [1]. The disc contacts with fibrocartilaginous articular surfaces: the articular surface on the head of the mandible, the articular fossa, and the articular tubercle of the zygomatic process of the temporal bone [1,3,4].

The interior of the TMJ is filled with synovial fluid, which is derived from two sources: plasma and two types of synoviocytes: A and B [1]. Synovial fluid plays a crucial role in ensuring proper joint function, acting as a lubricant that reduces friction between joint surfaces. Due to its hyaluronan content, it exhibits viscoelastic properties that cushion mechanical loads transferred to joint cartilage. Additionally, it plays a trophic role by providing nutrients to the avascular cartilage, which is crucial for maintaining its integrity and metabolism.

From a clinical perspective, the condition of the synovial environment plays a key role in the susceptibility of the temporomandibular joint to infectious processes. Inflammation, whether secondary to systemic diseases, local trauma, or degenerative changes, can alter the composition of synovial fluid and impair its protective functions. Joint effusion, increased vascular permeability, and impaired synovial membrane integrity can facilitate the penetration and proliferation of microorganisms within the joint. These changes often manifest clinically as pain, swelling, increasing limitation of mouth opening, and impaired function.

An important component of synovial fluid that can serve as a nutrient for bacteria is glucose—an important energy substrate for many metabolic processes of microorganisms. Glucose enters synovial fluid from blood plasma through the semipermeable synovial membrane. Under physiological conditions, its concentration in synovial fluid is similar to blood glucose levels, although slightly lower, as presented in Table 1. An increase in blood glucose levels also causes its increase in synovial fluid [5]. Bacteria present in the joint can use glucose as an energy source, which promotes their proliferation and increases the risk of infection. In patients with hyperglycemia, such as those associated with diabetes, increased glucose levels in synovial fluid can create particularly favorable conditions for the development of infection. Therefore, individuals with elevated blood glucose levels are at greater risk of developing SATMJ [6].

### 1.2. Rationale

Septic temporomandibular joint disease (SATMJ) is a rare and poorly understood condition, most often described in isolated cases. Despite not being indexed in Orphanet (EU) and Genetic and Rare Diseases (USA), SATMJ appears to more than meet the criteria for a rare disease, but epidemiological studies are lacking to confirm this. There is currently no systematic understanding of its etiology and risk factors. Conducting a systematic review will fill this gap and provide the data necessary for improved diagnosis and treatment of SATMJ.

### 1.3. Objectives

The aim of this study was to verify two hypotheses regarding septic arthritis of the temporomandibular joint (SATMJ), which were established in a previously published protocol [8]. The first hypothesis (H_0_-1) assumes that the occurrence of SATMJ is not related to gender, age, race, comorbidities, immunological status, or general health status of the patient. The second hypothesis (H_0_-2) concerns the route of spread of infection to the temporomandibular joint and assumes the lack of dominance of any of the possible routes, including otogenic, odontogenic, or other mechanisms. The study aimed to assess the statistical significance of the associations between these factors and the development of SATMJ.

## 2. Methods

The protocol for this review has previously been published as a separate journal article [8]. This review has been registered on the PROSPERO International Prospective Register of Systematic Reviews. PROSPERO registration ID: CRD42024613462.

The methods are briefly presented below. Their more detailed description can be found in the protocol [8].

### 2.1. Eligibility Criteria

Patient selection followed a published protocol [8]. Included were individuals with TMJ inflammation diagnosed clinically and with at least one identified etiological factor, such as a risk factor or a pathogen causing inflammation. Excluded were animal, cadaveric, pediatric, and unpublished studies, including preprints and conference proceedings. All TMJ-targeted interventions were included, excluding systemic-only treatments. While no formal language restrictions were applied during the search, only articles available in English were found to be eligible. Comparators were not required; case series were disaggregated into individual cases and analyzed separately. Key outcomes included demographics, risk factors, and microorganisms, as well as diagnostic methods and treatment-related outcomes. The eligibility criteria are summarized in Table 2.

### 2.2. Information Sources

According to our previously published protocol, the following databases are included in the review: ACM, BASE, CENTRAL, Scopus, ClinicalTrials.gov, PubMed, and Embase. Gray literature was searched using Google Scholar. Reference lists were also searched. No language or time restrictions are imposed. The selected databases cover a wide range of sciences and contain peer-reviewed publications. Although no time restrictions were applied during the literature search, the studies included in the final analysis were published between 1955 and 2024.

### 2.3. Search Strategy

Based on initial searches using the words “temporomandibular”, “septic”, and “arthritis” and previously established criteria, a detailed query was developed. The priority was to maximize the capture of primary studies, even at the cost of increasing the number of results for screening. The final version of the query was “(temporomandibular OR temporomandibularis OR TMJ) AND (joint OR articulatio) AND (arthritis OR inflammation OR inflammatory OR infection OR infections OR empyema OR abscess OR osteomyelitis) AND (septic OR suppurative OR pyogenic OR microbial OR bacterial OR Gram-positive OR staphylococcal OR fungal OR viral)”. The last search was performed on 14 August 2024.

### 2.4. Selection Process

Search results were imported into the Rayyan automation tool (version 2025-07-10, Qatar Computing Research Institute, Doha, Qatar, and Rayyan Systems, Cambridge, MA, USA) and then manually duplicated. Titles and abstracts were reviewed blindly by two independent reviewers (K.L. and K.G.). In case of discrepancies, publications were considered for further review. Cohen’s kappa was calculated for consistency. Full-text review was then performed; in case of any disagreements, a third reviewer (M.C.) was included, and the final decision was made. During the article selection process, two studies were excluded because they did not identify an etiological factor (Ayachi 2016 [9]; Azmi 2021 [10]). The reasons for exclusion were in accordance with the adopted methodological criteria described in the “Inclusion and Exclusion Criteria” section. The entire process was illustrated in the completed PRISMA 2020 flowchart in Supplementary Materials.

### 2.5. Data Collection Process

Data was collected independently by two researchers (K.L. and K.G.), and in case of discrepancies, the decision was made by a third researcher (M.C.). Results are presented in a table using Google Workspace (version 2025-07-15, Google LLC, Mountain View, CA, USA).

### 2.6. Data Items

In order to identify the factors determining the development of SATMJ, demographic data, information on systemic and local diseases, results of microbiological tests, and routes of spread of infection were collected and analyzed. Bacteria were classified as aerobic, anaerobic, or facultative anaerobic based on descriptions in the original case reports. When not explicitly stated, classification was assigned manually using standard microbiological references. Mixed infections were included in both relevant categories. Any uncertainties were resolved by reviewer consensus. These data, treated as results of the interview, physical examination, and additional tests, allowed us to determine the characteristics of the group of patients. Their systematic comparison allowed for the identification of subgroups, e.g., assessing the relationship between skin injuries and bacterial infections or between age and the spread of odontogenic infection to the temporomandibular joint.

### 2.7. Study Risk of Bias Assessment

Two independent authors (K.L. and K.G.) assessed the risk of bias using quality assessment tools developed by the Joanna Briggs Institute (JBI). The JBI critical appraisal tools for case reports and case series were applied systematically and in line with official guidance. Many included studies had incomplete reporting, which limited the available information. Where reporting was incomplete, no assumptions were made; instead, such items were coded as ‘unclear’ to enhance transparency and reproducibility. The quality scores reflect both methodological rigor and reporting depth.

Specifically, tools for case series and single case reports were used. In case of discrepancies, a third investigator (M.C.) joined the assessment, and the final decision was made by secret ballot. 

For case series, 10 closed questions (yes/no) were provided, concerning, among others, clear inclusion criteria, reliable diagnostic methods, completeness of data, demographic information, and adequacy of statistical analysis.

For single case descriptions, 8 questions were used, covering, among others, patient demographic data, description of medical history, clinical condition, interventions performed, effects of treatment, and practical conclusions.

For each report, a summary of the number of affirmative responses was prepared, as well as detailed tables with answers to each question. A quantitative assessment was also made: on a scale of 0–10 (for case series) and 0–8 (for case reports). The exact course and details of this process can be found in the previously published protocol [8]. All papers, regardless of the Risk of Bias rating, were included in the synthesis due to the small number of identified studies.

### 2.8. Statistical Methods

Statistical analyses include determinants, treatment methods, and outcomes. According to the study protocol, the statistical analysis was intended to include: (1) measures of central tendency (mean, median), (2) measures of dispersion (range, standard deviation), (3) frequency (percentages and counts), (4) relationships (correlations and regressions, e.g., age), (5) visualizations (histograms, scatter plots with trend lines). Correlation and regression analyses were not performed due to insufficient and ineligible data.

Due to the small sample size and dichotomous nature of the data, we refrained from analyzing Spearman and Pearson correlations, as planned in the protocol, which require continuous data or large samples. Instead, we used methods appropriate to the scale of measurement and sample size, enabling us to reliably determine the relationship between variables. The percentage of individual etiological factors present in the patients was calculated. The mean, median, and standard deviation were also calculated.

Results were presented graphically, with a significance level of α = 0.05. MedCalc (version 23.3.5, MedCalc Software, Ostend, Belgium) and Google Workspace (version 2025-07-15, Google LLC, Mountain View, CA, USA) software were used.

### 2.9. Certainty Assessment

The certainty of the evidence was assessed using the GRADE tool, which classifies evidence into four quality levels and rates the strength of recommendations as strong or weak, taking into account factors such as the risk of bias, consistency, and precision of results.

## 3. Results

**Figure 1:**
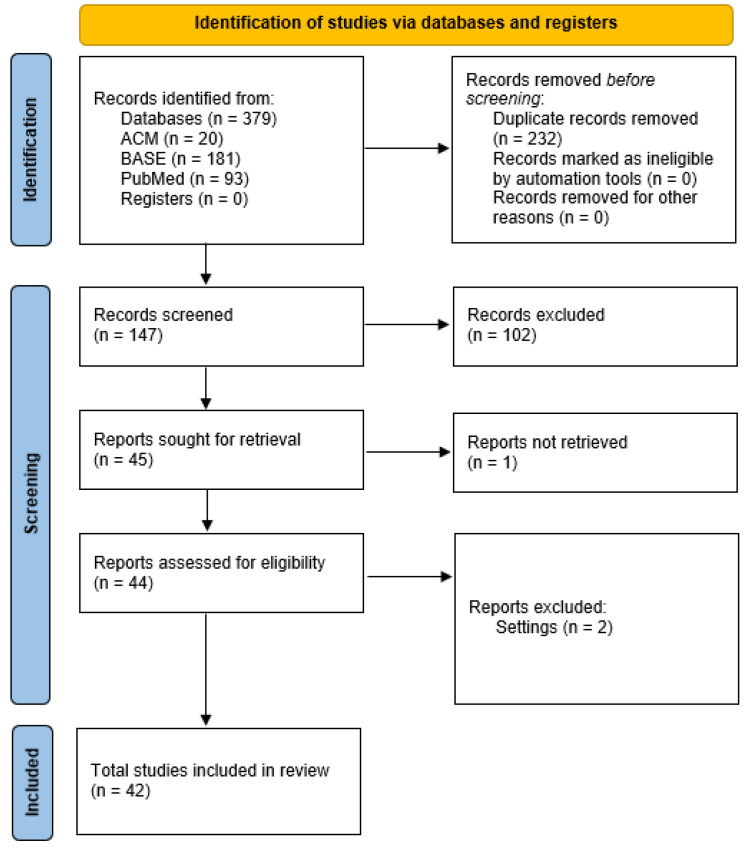
PRISMA flow diagram.

The individual stages of the results presentation are illustrated below in the flow diagram and tables (Figure 1, Table 3).

### 3.1. Study Selection

**Figure 2:**
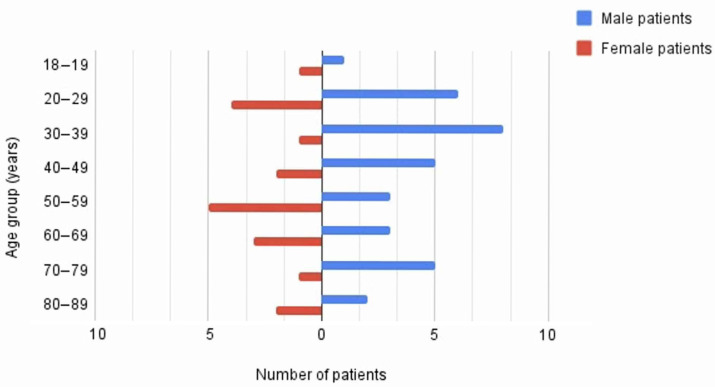
Age distribution of patients.

Cohen’s κ was about 0.960, indicating almost perfect consistency between reviewers. The selection is presented as a flow diagram in Figure 2.

### 3.2. Study Characteristics

The table below (Table 3) presents the summary of study characteristics.

Detailed characteristics of individual studies and patients are provided in Appendix A in Table A1.

### 3.3. Risk of Bias in Studies

The summary of risk of bias in qualified case series and case reports is presented in the table below (Table 4).

Risk of bias was assessed using design-specific tools (JBI checklist for case reports and case series). Detailed item-level assessments for individual studies are provided in Appendix A in Table A2 and Table A3.

### 3.4. Results of Individual Studies

In the analyzed cases of SATMJ, anaerobic bacteria predominated in 33 cases, while aerobic infections were associated with 21 cases. Gram-positive infections occurred in 40 cases and Gram-negative infections in 13 cases. Fungal agents were identified in five cases. Odontogenic infections were reported in 12 cases, and previous surgical procedures or trauma occurred in eight patients. Seven cases were associated with local non-odontogenic infections. Among the patients, seven had diabetes, five had immunodeficiencies, and concomitant systemic diseases were present in 12 cases. Complete patient data, including age, gender, and detailed characteristics of the infection, are presented in Appendix A in Table A4.

### 3.5. Result of Syntheses

Clinical and demographic data consists of 59 subjects, including 36 men and 23 women, aged 18 to 87, with a mean age of approximately 47 years (SD = 20; median = 46). Data were coded binary (0/1) to indicate the presence or absence of various infections and diseases. The most common infections were anaerobic or facultative anaerobic infections (78% [46/59], 95% CI: 65.3–87.7%). Gram-positive infections (73% [43/59], 95% CI: 59.7–83.6%) were more common than Gram-negative infections (37% [22/59], 95% CI: 25.0–50.9%). Aerobic infections were less common (30%, 95% CI: 19.2–43.9%). Other clinical features, such as fungal infections, odontogenic problems, diabetes, immune deficiency, and systemic diseases, were observed in 2% (95% CI: 0.0–9.1%) to 7% (95% CI: 1.9–16.5%) of patients. Previous surgical procedures or trauma and local non-odontogenic infections were also noted in 5% (95% CI: 1.1–14.1%) of cases. Due to the type of papers examined (case reports, case series), individual etiological factors were assessed as “Very low certainty” according to the GRADE assessment.

The distribution of male and female patients across different age groups was analyzed (Figure 2). It shows a bimodal pattern for both sexes, but with notable differences. Male patients, marked in blue, are most concentrated in the 20–29 and 30–39 age groups, with a visible peak in the latter. In contrast, female patients, marked in red, are more widely distributed, with notable numbers in the 20–29, 50–59, and 60–69 age groups. Younger age groups (under 20) and the oldest groups (80–99) have relatively fewer patients overall. The 30–39 age group has the highest number of male patients, while the 50–59 age group has the highest number of female patients. This points to age-related and possibly condition-related differences in the sex distribution of the patient population.

When analyzing the relationship between various risk factors and types of infections, certain associations between variables are noticeable. Age and gender do not have a strong influence on the risk of infection, although some associations may exist in the context of other factors. Anaerobic or facultative anaerobic infections are usually inversely related to aerobic infections, suggesting that the presence of one reduces the likelihood of the other. Infections with Gram-negative and Gram-positive bacteria rarely co-occur. Fungal infections, on the other hand, are more closely associated with a history of surgery or trauma, indicating an increased risk of this type of infection in these cases. Odontogenic infections may also have a possible association with the presence of other types of infections, especially local ones. Furthermore, people with diabetes are more susceptible to infections, including those related to the respiratory system. Immunosuppression also increases the risk of infection. Finally, systemic diseases have some influence on the risk of infection, but their influence on other factors is limited. The conclusions presented above indicate varying levels of infection risk depending on factors such as medical history, type of infection, and presence of diabetes.

The presented relationships provide valuable data allowing for the identification of links between patient characteristics and the nature of infections. This allows for the separation of clinical phenotypes with a different risk of infection and a potentially diverse clinical course, which in the future may be the basis for the implementation of a personalized diagnostic and therapeutic approach. Given the very low GRADE certainty for most factors, these patterns should be considered exploratory and interpreted cautiously.

## 4. Discussion

### 4.1. General Interpretation of the Results

The analysis of data from patients with temporomandibular joint (SATMJ) inflammation revealed potential associations between the type of infection, comorbidities, demographic characteristics, and possible molecular indicators of infection. It shows that age and gender have minimal impact on the risk of infection. Fungal infections are associated with a history of surgery or trauma, and dental problems increase the risk of other infections. Diabetes, immunosuppression, and systemic diseases also influence risk, although to varying degrees.

Diabetes, in particular, is clearly associated with an increased incidence of temporomandibular joint infection [11,12]. Hyperglycemia leads to elevated glucose concentrations in synovial fluid, which favors the growth of pathogens and can alter the molecular composition of the synovial fluid, increasing its susceptibility to bacterial colonization [13,14]. Furthermore, diabetes is associated with immune dysfunction, including impaired phagocytosis, chemotaxis, and inflammatory response, as well as microangiopathy that limits tissue perfusion [15,16]. All of these factors can lead to a more severe and recalcitrant course of SATMJ in diabetic patients.

In the context of demographic variables, both age and gender showed only limited associations with other clinical factors. However, the analysis of the distribution of patients by age and gender revealed a clear trend—the younger age groups (20–39 years) were dominated by men, while in the older groups (50–69 years) women predominated. This may suggest differences in the risk factors for the development of SATMJ between the sexes, potentially related to different immunological, hormonal, or lifestyle factors. It is also worth noting that elderly patients (70+ years), regardless of gender, were present in the analysis, which indicates that SATMJ may also occur in older people, especially in the context of comorbid systemic diseases. Comprehensive assessment of relationships between the occurrence of etiological factors and demographic characteristics provides valuable information on possible mechanisms of development and risk factors of SATMJ, which may be of clinical importance in the diagnosis and treatment of this rare but serious condition.

### 4.2. Strengths

The strength of this research is its comprehensive nature, combining clinical, microbiological, and demographic analysis of patients with SATMJ. An additional advantage is the inclusion of demographic variables, such as age and gender, which allowed us to identify interesting epidemiological trends, especially differences between sexes in different age groups. This study contributes valuable knowledge to the problem of SATMJ, which is rarely described in medical literature, and provides a solid basis for designing further, more advanced prospective studies.

### 4.3. Limitations

This study has several important limitations that should be considered when interpreting the results. First, the sample size was limited, which narrowed the possibilities of statistical analysis. The analysis was retrospective, which is associated with the risk of errors in medical records and incomplete clinical data.

The study also did not take into account other potential confounding factors, such as socioeconomic status, treatment, or duration of symptoms before diagnosis. In the case of demographic variables, such as age and gender, limitations related to unequal representation across age groups may distort the observed trends. Additionally, the lack of microbiological and molecular data on the exact mechanism of drug resistance or bacterial strains limits the possibility of a deeper interpretation of the relationship between the type of infection and comorbidities. For this reason, the obtained results should be interpreted with caution and treated as a preliminary to further, more detailed prospective studies on larger groups of patients.

### 4.4. Applicability and Future Research

Given the limitations described above, future studies should be prospective and include larger, more diverse patient populations. It is worth considering the use of more advanced statistical analysis methods to more precisely determine the role of individual factors in the pathogenesis of SATMJ. Additionally, expanding the research to include a molecular and microbiological component, such as pathogen DNA sequencing or study of treatment resistance, could contribute to a better understanding of the disease mechanisms and the development of more effective therapeutic strategies.

## 5. Conclusions

The collected data indicate a clinical pattern dominated by anaerobic and Gram-positive infections, probably of odontogenic origin. However, there is also a smaller group of cases caused by Gram-negative infections, which most often coexist with anaerobic bacteria. It appears that additional systemic health burdens may increase the susceptibility of patients to SATMJ, as suggested by strong connections of non-odontogenic infections with diabetes, systemic diseases, and immunoincompetence. The present findings are consistent with the possibility that general health and immune status of the patient may have a significant impact on the occurrence of SATMJ, which challenges hypothesis H_0_-1. At the same time, the observations regarding the route of spread of infection—with a predominance of odontogenic cases, but also with the involvement of other mechanisms—point towards the possibility of a dominant route of infection, which, if confirmed, could challenge hypothesis H_0_-2.

## Figures and Tables

**Table 1:** Comparison of glucose levels in blood and synovial fluid as reported in the studies on periprosthetic joint infection [7].

Condition	Glucose Concentration in Blood (mg/dL)	Glucose Concentration in Synovial Fluid (mg/dL)
Euglycemia	70–100	60–95
Mild hyperglycemia (prediabetes)	100–125	90–120
Hyperglycemia (diabetes)	≥126	115–135

**Table 2:** Eligibility criteria.

	Criteria for Inclusion	Criteria for Exclusion
Patients	SATMJ cases with at least one risk factor or pathogen causing inflammation identified	Animal studies, cadaver studies, pediatric patients,
Intervention	Any conservative or surgical treatment	Treatment of a systemic disease only
Comparison	Not required	Not applicable
Outcomes—etiological factors	Demographics, systemic disease, local injury or disease, microbiological agent	Not applicable
Outcomes—diagnostics and treatment	Diagnostic methods used, conservative treatment, invasive treatment, hospitalization length, resolution and recurrences, complications	Not applicable
Settings	Primary studies, e.g., case series, case reports	Preprints, conference proceedings, book chapters,

**Table 3:** Summary of study characteristics.

Characteristic	Summary of Findings
Number of included publications	42
Total number of reported cases	50+ (case reports and case series)
Publication years	1955–2024
Age (range)	18–87 years
Sex distribution	Predominantly male
Affected temporomandibular joint	Unilateral involvement in all reported cases
Most frequently isolated pathogens	*Staphylococcus aureus* (most common), other *Staphylococcus* spp., *Streptococcus* spp.
Gram-positive infections	Predominant
Gram-negative infections	Less frequent (e.g., *Pseudomonas*, *Klebsiella*, *Escherichia coli*)
Fungal infections	Rare (mainly *Aspergillus* spp.)
Reported systemic diseases	Diabetes mellitus most frequent; malignancy and immunosuppression occasionally reported
Ear-related infections	Reported in a minority of cases (e.g., otitis media/externa)
Odontogenic or oropharyngeal source	Identified in a subset of cases
Previous surgery or trauma	Occasionally reported
Oral health–related factors	Sporadically reported
Other predisposing factors	Rare and heterogeneous

**Table 4:** The summary of risk of bias assessment.

Study Design	Low Risk	Moderate Risk	High Risk	Total
Case reports	22 (61%)	10 (28%)	4 (11%)	36
Case series	1 (17%)	1 (17%)	4 (66%)	6

## Data Availability

The original contributions presented in this study are included in the article. The case-level coded dataset used for the analysis is available from the corresponding author upon reasonable request.

## Supplementary Materials

The following supporting information can be downloaded at: https://www.mdpi.com/article/10.3390/jcm15020706/s1, PRISMA_2020_abstract_checklist; PRISMA 2020 Checklist [17].

## Appendix A

**Table A1:** Study characteristics.

First Author, Publication Year	DOI	Age	Sex	Affected TMJs	Presented Bacteria	Systemic Disease	Ear Diseases	Preauricular Area Disease, Injury, or Treatment	Surgical Treatment	Oral Health Issues	Other Factors
Al-Khalisy, 2015 [18]	https://doi.org/10.4103/1947-2714.168678	24	M	1	Coagulase-negative *Staphylococcus*	N/S	N/A	N/S	N/S	N/A	N/A
Ângelo, 2023 [19]	https://doi.org/10.1016/j.ijom.2023.06.008	68	M	1	*Pseudomonas aeruginosa*	Diabetes, immunosuppressive treatment	N/A	Chronic purulent otitis media	N/S	N/A	N/A
Araz Server, 2017 [20]	https://doi.org/10.1016/j.ijom.2017.04.007	55	F	1	*Staphylococcus haemolyticus*	Diabetes	N/A	N/S	N/S	Peritonsillar abscess, Tonsillitis	N/A
Baniel, 2020 [21]	https://doi.org/10.1016/j.amjmed.2019.07.006	85	F	1	MRSA	N/S	N/A	N/S	N/S	N/A	N/A
Bounds, 1987 [22]	https://doi.org/10.1016/0266-4356(87)90158-6	72	F	1	*Staphylococcus aureus*	N/S	N/A	Fracture of the posterior wall of the temporomandibular joint into the external auditory meatus	N/S	N/A	Chin trauma
Cai, 2010 [23]	https://doi.org/10.1016/j.joms.2009.07.060	48	M	1	Gram-positive *coccus*	N/S	N/A	N/A	N/A	N/A	N/A
Cai, 2010 [23]	https://doi.org/10.1016/j.joms.2009.07.060	26	F	1	*Staphylococcus saprophyticus*	N/S	N/A	N/A	N/A	N/A	N/A
Cai, 2010 [23]	https://doi.org/10.1016/j.joms.2009.07.060	22	F	1	Gram-positive *coccus*	N/S	N/A	N/A	N/A	N/A	N/A
Cai, 2010 [23]	https://doi.org/10.1016/j.joms.2009.07.060	30	M	1	Gram-positive *coccus*	N/S	N/A	N/A	N/A	N/A	N/A
Cai, 2010 [23]	https://doi.org/10.1016/j.joms.2009.07.060	19	F	1	Gram-positive *coccus*	N/S	N/A	N/A	N/A	N/A	N/A
Cai, 2010 [23]	https://doi.org/10.1016/j.joms.2009.07.060	46	M	1	*Staphylococcus saprophyticus*	N/S	N/A	N/A	N/A	N/A	N/A
Cai, 2010 [23]	https://doi.org/10.1016/j.joms.2009.07.060	25	M	1	*Staphylococcus aureus*	N/S	N/A	N/A	N/A	N/A	N/A
Cai, 2010 [23]	https://doi.org/10.1016/j.joms.2009.07.060	54	M	1	N/S	N/A	N/A	Osteoarthritis	N/A	N/A	N/A
Cai, 2010 [23]	https://doi.org/10.1016/j.joms.2009.07.060	21	F	1	N/S	N/A	N/A	Fibrosis	N/A	N/A	N/A
Cai, 2010 [23]	https://doi.org/10.1016/j.joms.2009.07.060	45	M	1	Gram-positive *coccus*	N/A	N/A	N/A	N/A	N/A	N/A
Cai, 2010 [23]	https://doi.org/10.1016/j.joms.2009.07.060	40	F	1	*Staphylococcus saprophyticus*	N/A	N/A	N/A	N/A	N/A	N/A
Cai, 2010 [23]	https://doi.org/10.1016/j.joms.2009.07.060	37	M	1	*Staphylococcus aureus*	N/A	N/A	N/A	N/A	N/A	N/A
Cai, 2010 [23]	https://doi.org/10.1016/j.joms.2009.07.060	63	M	1	Gram-positive *coccus*	N/A	N/A	N/A	N/A	N/A	N/A
Cheong, 2017 [24]	https://doi.org/10.1155/2017/3641642	76	M	1	*Achromobacter xylosoxidans*	N/A	Acute otitis media	N/A	N/A	N/A	N/A
Dias Ferraz, 2021 [25]	https://doi.org/10.1080/08869634.2019.1661943	64	F	1	*Pseudomonas* spp.	N/A	N/A	Mandible angle fracture	N/A	N/A	N/A
Dias Ferraz, 2021 [25]	https://doi.org/10.1080/08869634.2019.1661943	34	M	1	*Staphylococcus* *aureus*	N/A	N/A	N/A	N/A	Odontogenic abscess, III molar extraction	N/A
Dias Ferraz, 2021 [25]	https://doi.org/10.1080/08869634.2019.1661943	21	M	1	*Streptococcus* group D	Asthma	N/A	N/A	N/A	Odontogenic abscess	N/A
Døving, 2021 [26]	https://doi.org/10.1007/s10006-020-00921-z	72	M	1	*Streptococcus constellatus* *Dialister pneumosintes*	N/A	N/A	N/A	N/A	N/A	N/A
Gams, 2016 [27]	https://doi.org/10.1016/j.joms.2015.11.003	25	M	1	Bacteroides uniformis	Diabetes	N/A	N/S	N/A	Odontogenic infection	N/A
Gayle, 2013 [28]	https://doi.org/10.1016/j.jemermed.2013.01.034	18	F	1	*Staphylococcus*, Group C *Streptococcus*, and *Acinetobacter baumannii*	N/A	N/A	N/A	N/A	N/A	N/A
Granadas, 2023 [29]	https://doi.org/10.35100/eurorad/case.18139	59	F	1	*Staphylococcus aureus*	N/A	N/A	N/A	N/A	N/A	N/A
Hincapie, 1999 [30]	https://doi.org/10.1053/hn.1999.v121.a96115	33	M	1	*Klebsiella pneumoniae*	N/A	N/A	N/A	N/A	N/A	N/A
Ishikawa, 2017 [31]	https://doi.org/10.1007/s10006-016-0604-z	87	M	1	MRSA	N/A	N/A	N/A	N/A	N/A	N/A
Kim, 2011 [32]	https://doi.org/10.5125/jkaoms.2011.37.6.510	46	M	1	*Alpha streptococci* species	N/A	N/A	N/A	N/A	N/A	N/A
Kim, 2011 [32]	https://doi.org/10.5125/jkaoms.2011.37.6.510	43	F	1	*Alpha streptococci* species	N/A	N/A	N/A	N/A	N/A	N/A
Kim, 2019 [33]	https://doi.org/10.14476/jomp.2019.44.3.127	49	M	1	*Haemophilus aphrophilus*	Diabetes	N/A	N/S	Trigeminal nerve block treatment	N/A	N/A
Klüppel, 2012 [34]	https://doi.org/10.1097/SCS.0b013e3182646061	58	F	1	*Staphylococcus aureus*	N/A	N/A	N/A	N/A	N/A	N/A
Levorova, 2017 [35]	https://doi.org/10.1016/j.ijom.2016.09.008	38	F	1	*Raoultella ornithinolytica*	N/A	N/A	N/A	N/A	N/A	N/A
Lohiya, 2016 [36]	https://doi.org/10.1016/j.joms.2015.06.166	56	F	1	Coagulase-negative *Staphylococcus aureus*	N/A	N/A	N/A	N/A	N/A	N/A
Lohiya, 2016 [36]	https://doi.org/10.1016/j.joms.2015.06.166	49	M	1	MRSA	N/A	N/A	N/A	N/A	N/A	N/A
Moses, 1998 [37]	https://doi.org/10.1016/S0278-2391(98)90725-X	36	M	1	*Streptococcus anginosus*, *Streptococcus viridans*,	N/A	N/A	N/A	N/A	N/A	N/A
Nagarakanti, 2020 [38]	https://doi.org/10.1177/0145561320944648	58	M	1	MSSA *Candida glabrata*	N/A	N/A	N/A	N/A	N/A	N/A
Oliveira, 2024 [39]	https://doi.org/10.7759/cureus.64480	61	F	1	*Staphylococcus aureus*	N/A	N/A	N/A	N/A	N/A	N/A
Sembronio, 2007 [40]	https://doi.org/10.1016/j.tripleo.2006.08.028	52	F	1	*Streptococcus* sp.	N/A	N/A	N/A	N/A	N/A	N/A
Suda, 2017 [41]	https://doi.org/10.4236/ojst.2017.74018	85	M	1	*Propionibacterium* spp. *Veillonella* spp.	N/A	N/A	N/A	N/A	N/A	N/A
Thakur, 2022 [42]	https://doi.org/10.1136/bcr-2021-247111	Late 30 s	M	1	*Klebsiella pneumoniae*	N/A	N/A	N/A	N/A	N/A	N/A
Thomson, 1989 [43]	https://doi.org/10.1017/s0022215100108813	59	F	1	*Staphylococcus* *aureus*	N/A	N/A	N/A	N/A	N/A	N/A
Thomson, 1989 [43]	https://doi.org/10.1017/s0022215100108813	70	M	1	*Staphylococcus* *aureus*	N/A	N/A	N/A	N/A	N/A	N/A
Trimble, 1983 [44]	https://doi.org/10.1016/S0301-0503(83)80022-8	53	F	1	*Staphylococcus* *aureus*	N/A	N/A	N/A	N/A	N/A	N/A
Turton, 2022 [45]	https://doi.org/10.1007/s12663-021-01637-7	18	M	1	*Fusobacterium necrophorum*	N/A	N/A	N/A	N/A	N/A	N/A
Varghese, 2015 [46]	PMID: 25738723	74	M	1	*Aspergillus flavus*	N/A	N/A	N/A	N/A	N/A	N/A
Xiao, 2017 [47]	https://doi.org/10.3892/etm.2017.4510	83	F	1	*Escherichia coli*	N/A	N/A	N/A	N/A	N/A	N/A
Yang, 2016 [48]	https://doi.org/10.5125/jkaoms.2016.42.4.227	52	M	1	*Staphylococcus aureus*	N/A	N/A	N/A	N/A	N/A	N/A
Metzger, 1970 [49]	https://doi.org/10.7326/0003-4819-73-2-267-	23	M	1	*Neisseria gonorrhoeae*	Mumps	N/A	N/A	N/A	N/A	Sore throat
Alexander, 1973 [50]	https://doi.org/10.1016/0030-4220(73)90331-9	21	M	1	*Neisseria gonorrhoeae*	N/A	N/A	N/A	N/A	N/A	Granuloma, hard drug use
Winters, 1955 [51]	https://doi.org/10.1016/0030-4220(55)90185-7	19	M	1	*Staphylococcus albus*	N/A	N/A	N/A	N/A	N/A	N/A
Seymour, 1982 [52]	https://doi.org/10.1016/S0007-117X(82)80021-8	19	F	1	*Haemophilus influenzae*	Arthritis	N/A	N/A	N/A	N/A	Upper respiratory tract infection
Goldschmidt, 2002 [53]	https://doi.org/10.1053/joms.2002.35736	35	M	1	Group A beta-hemolytic *Streptococcus*	N/A	N/A	N/A	N/A	N/A	Pharyngitis, HBV, HCV, cocaine user, venereal disease
Enomoto, 2012 [54]	https://doi.org/10.1016/j.joms.2011.06.215	70	M	1	*Prevotella*, *Haemophilus*, *Streptococcus*	Prostate carcinoma	N/A	N/A	N/A	Pericoronitis, periapical lesion of II/III mola	Bisphosphonates therapy
Vongsfak, 2020 [55]	https://doi.org/10.1016/j.inat.2020.100709	65	F	1	*Staphylococcus aureus*	N/A	N/A	N/A	N/A	N/A	Subgaleal abscess
Noroy, 2020 [56]	https://doi.org/10.1016/j.anorl.2019.09.010	68	M	1	*Aspergillus flavus*, *Staphylococcus epidermidis*	Diabetes	Otitis externa	N/A	N/A	N/A	Papillary renal cell carcinoma
Chue, 1975 [57]	https://doi.org/10.1016/0030-4220(75)90198-X	27	F	1	*Neisseria gonorrhoeae*	N/A	N/A	N/A	N/A	N/A	Acute gonorrhea
Murakami, 1984 [58]	https://doi.org/10.1016/s0301-0503(84)80209-x	43	M	1	N/A	N/A	N/A	N/A	N/A	Periapical lesion	N/A
Hilbert, 1984 [59]	https://doi.org/10.1016/0305-4179(84)90032-9	37	M	1	N/A	N/A	Otitis externa	N/A	N/A	N/A	24% third degree burns

MRSA—Methicillin-resistant *Staphylococcus aureus*; MSSA—Methicillin-sensitive *Staphylococcus aureus*; N/A—not applicable; N/S—not specified.

**Table A2:** Risk of bias in case series assessment.

First Author, Publication Year	1.	2.	3.	4.	5.	6.	7.	8.	9.	10.	Favorable (“Yes”) Answers
Thomson, 1989 [43]	No	Unclear	No	No	No	Unclear	Yes	Yes	No	N/A	2
Kim, 2011 [32]	No	Yes	No	No	No	Unclear	No	Yes	No	N/A	2
Lohiya, 2016 [36]	No	Yes	No	No	No	Unclear	Yes	Yes	No	N/A	3
Gayle, 2013 [28]	No	Yes	No	No	No	Unclear	Yes	Yes	No	N/A	3
Dias Ferraz, 2021 [25]	No	Yes	No	Yes	Yes	Yes	No	Yes	No	N/A	5
Cai, 2010 [23]	No	Yes	No	Yes	Yes	Yes	Yes	Yes	Yes	Yes	9

N/A—not applicable. 1. Were there clear criteria for inclusion in the case series? 2. Was the condition measured in a standard, reliable way for all participants included in the case series? 3. Were valid methods used for identification of the condition for all participants included in the case series? 4. Did the case series have consecutive inclusion of participants? 5. Did the case series have complete inclusion of participants? 6. Was there clear reporting of the demographics of the participants included in the study? 7. Was there clear reporting of clinical information of the participants? 8. Were the outcomes or follow-up results of cases clearly reported? 9. Was there clear reporting of the presenting sites’/clinics’ demographic information? 10. Was statistical analysis appropriate?

**Table A3:** Risk of bias in case reports assessment.

First Author, Publication Year	1.	2.	3.	4.	5.	6.	7.	8.	Favorable (“Yes”) Answers
Al-Khalisy, 2015 [18]	Yes	Yes	Yes	Yes	Unclear	No	Yes	Yes	6
Ângelo, 2023 [19]	Yes	Yes	Yes	Yes	Yes	Yes	Yes	Yes	8
Sembronio, 2007 [40]	Yes	Yes	Yes	Yes	Yes	Yes	No	Yes	7
Hincapie, 1999 [30]	Yes	Yes	Yes	Yes	Unclear	Unclear	No	Yes	5
Moses, 1998 [37]	Yes	Yes	Yes	Yes	Yes	Unclear	Yes	Yes	7
Døving, 2021 [26]	Yes	Yes	Yes	Yes	Yes	Yes	No	Yes	7
Gams, 2016 [27]	Yes	Yes	Yes	Yes	Yes	Yes	No	Yes	7
Granadas, 2023 [29]	No	Unclear	Unclear	Yes	Unclear	No	No	Yes	2
Ishikawa, 2017 [31]	Yes	Yes	Unclear	Yes	Unclear	Yes	No	Yes	5
Levorova, 2017 [35]	Yes	No	Yes	Yes	Yes	Yes	Yes	Yes	7
Kim, 2019 [33]	Yes	Yes	Yes	Yes	Unclear	Yes	No	Yes	6
Klüppel, 2012 [34]	Yes	Yes	Yes	Yes	Yes	Yes	No	Yes	7
Nagarakanti, 2020 [38]	Yes	Yes	Yes	Yes	Yes	Yes	No	No	6
Oliveira, 2024 [39]	Yes	Yes	Yes	Yes	Unclear	Yes	No	Yes	6
Araz Server, 2017 [20]	Yes	Yes	Yes	Yes	Yes	Yes	No	Yes	7
Suda, 2017 [41]	Yes	Yes	Yes	Yes	Yes	Yes	No	Yes	7
Yang, 2016 [48]	Yes	Yes	Yes	Yes	Yes	Yes	No	Yes	7
Varghese, 2015 [46]	Yes	Yes	Yes	Yes	Yes	Yes	Yes	Yes	8
Baniel, 2020 [21]	Yes	Yes	Yes	Yes	Yes	Yes	No	Yes	7
Bounds, 1987 [22]	Yes	Unclear	Unclear	Yes	Unclear	Yes	No	Yes	4
Cheong, 2017 [24]	Yes	Yes	Unclear	Yes	Yes	Yes	Yes	Yes	7
Thakur, 2022 [42]	Yes	Yes	Yes	Yes	Yes	Yes	No	Yes	7
Trimble, 1983 [44]	Yes	Yes	Yes	Yes	Unclear	Yes	No	Yes	6
Turton, 2022 [45]	Yes	Yes	Yes	Yes	Yes	Yes	No	Yes	7
Xiao, 2017 [47]	Yes	Yes	Yes	Yes	Yes	Yes	No	Yes	7
Murakami, 1984 [58]	No	Yes	Yes	Yes	Unclear	Unclear	No	Yes	4
Hilbert, 1984 [59]	Yes	Yes	Yes	Yes	Yes	Yes	No	Yes	7
Metzger, 1970 [49]	Yes	Yes	Yes	Yes	Yes	Yes	No	Yes	7
Winters, 1955 [51]	Yes	Yes	Yes	Yes	Yes	Unclear	Unclear	Yes	6
Alexander, 1973 [50]	Yes	Yes	Yes	Yes	Yes	Yes	Yes	Yes	8
Chue, 1975 [57]	Yes	Yes	Yes	Yes	Yes	Yes	No	Yes	7
Seymour, 1982 [52]	Yes	Yes	Yes	Yes	Yes	Yes	Yes	Yes	8
Goldschmidt, 2002 [53]	Yes	Yes	Yes	Yes	Yes	Yes	Yes	Yes	8
Enomoto, 2012 [54]	Yes	Yes	Yes	Yes	Yes	Yes	Yes	Yes	8
Vongsfak, 2020 [55]	Yes	Yes	Yes	Yes	Yes	Yes	Unclear	Yes	7
Noroy, 2020 [56]	Yes	Yes	Yes	Yes	Yes	Yes	No	Yes	7

N/A—not applicable. 1. Were the patient’s demographic characteristics clearly described? 2. Was the patient’s history clearly described and presented as a timeline? 3. Was the current clinical condition of the patient on presentation clearly described? 4. Were diagnostic tests or assessment methods and the results clearly described? 5. Was the intervention(s) or treatment procedure(s) clearly described? 6. Was the post-intervention clinical condition clearly described? 7. Were adverse events (harms) or unanticipated events identified and described? 8. Does the case report provide takeaway lessons?

**Table A4:** Summary of research results.

First Author, Publication Year	Sex (F:0/M:1)	Age	Anaerobic or Relatively Anaerobic Infection (0/1)	Aerobic Infection (0/1)	Gram (−) Infection (0/1)	Gram (+) Infection (0/1)	Fungal SATMJ (0/1)	Odontogenic Problem (0/1)	Previous Surgery and/or Trauma (0/1)	Local, Non-Odontogenic Infection (0/1)	Diabetes (0/1)	Immunoincompetence (0/1)	Systemic Disease (0/1)
Al-Khalisy, 2015 [18]	1	24	Yes	No	No	Yes	No	No	No	No	No	No	No
Ângelo, 2023 [19]	1	68	No	Yes	Yes	No	No	No	No	Yes	Yes	Yes	No
Araz Server, 2017 [20]	0	55	Yes	No	No	Yes	No	No	No	Yes	Yes	No	No
Baniel, 2020 [21]	0	85	Yes	No	No	Yes	No	No	No	No	No	No	No
Bounds, 1987 [22]	0	72	Yes	No	No	Yes	No	No	Yes	No	No	No	No
Cai, 2010 [23]	1	48	Yes	No	No	Yes	No	No	No	No	No	No	No
Cai, 2010 [23]	0	26	Yes	No	No	Yes	No	No	No	No	No	No	No
Cai, 2010 [23]	0	22	Yes	No	No	Yes	No	No	No	No	No	No	No
Cai, 2010 [23]	1	30	Yes	No	No	Yes	No	No	No	No	No	No	No
Cai, 2010 [23]	0	19	Yes	No	No	Yes	No	No	No	No	No	No	No
Cai, 2010 [23]	1	46	Yes	No	No	Yes	No	No	No	No	No	No	No
Cai, 2010 [23]	1	25	Yes	No	No	Yes	No	No	No	No	No	No	No
Cai, 2010 [23]	1	54	N/S	N/S	N/S	N/S	No	No	No	No	No	No	Yes
Cai, 2010 [23]	0	21	N/S	N/S	N/S	N/S	No	No	No	No	No	No	Yes
Cai, 2010 [23]	1	45	Yes	No	No	Yes	No	No	No	No	No	No	No
Cai, 2010 [23]	0	40	Yes	No	No	Yes	No	No	No	No	No	No	No
Cai, 2010 [23]	1	37	Yes	No	No	Yes	No	No	No	No	No	No	No
Cai, 2010 [23]	1	63	Yes	No	No	Yes	No	No	No	No	No	No	No
Cheong, 2017 [24]	1	76	No	Yes	Yes	No	No	No	No	No	No	No	No
Dias Ferraz, 2021 [25]	0	64	No	Yes	Yes	No	No	No	Yes	No	No	No	No
Dias Ferraz, 2021 [25]	1	34	Yes	No	No	Yes	No	Yes	No	No	No	No	No
Dias Ferraz, 2021 [25]	1	21	No	Yes	No	Yes	No	Yes	No	No	No	No	Yes
Døving, 2021 [26]	1	72	Yes	Yes	Yes	Yes	No	No	No	No	No	No	No
Gams, 2016 [27]	1	25	Yes	No	Yes	No	No	Yes	No	No	Yes	No	No
Gayle, 2013 [28]	0	18	Yes	Yes	Yes	Yes	No	No	No	No	No	No	No
Granadas, 2023 [29]	0	59	Yes	No	No	Yes	No	No	No	No	No	No	No
Hincapie, 1999 [30]	1	33	Yes	No	Yes	No	No	No	No	No	No	No	No
Ishikawa, 2017 [31]	1	87	Yes	No	No	Yes	No	No	No	No	No	No	No
Kim, 2011 [32]	1	46	No	Yes	No	Yes	No	No	No	No	No	No	No
Kim, 2011 [32]	0	43	No	Yes	No	Yes	No	No	No	No	No	No	No
Kim, 2019 [33]	1	49	Yes	No	Yes	No	No	No	Yes	No	Yes	No	No
Klüppel, 2012 [34]	0	58	Yes	No	No	Yes	No	No	No	No	No	No	No
Levorova, 2017 [35]	0	38	Yes	No	Yes	No	No	No	No	No	No	No	No
Lohiya, 2016 [36]	0	56	Yes	No	No	Yes	No	No	No	No	No	No	No
Lohiya, 2016 [36]	1	49	Yes	No	No	Yes	No	No	No	No	No	No	No
Moses, 1998 [37]	1	36	Yes	Yes	Yes	Yes	No	No	No	No	No	No	No
Nagarakanti, 2020 [38]	1	58	Yes	No	No	Yes	Yes	No	No	No	No	No	No
Oliveira, 2024 [39]	0	61	Yes	No	No	Yes	No	No	No	No	No	No	No
Sembronio, 2007 [40]	0	52	No	Yes	No	Yes	No	No	No	No	No	No	No
Suda, 2017 [41]	1	85	Yes	No	Yes	Yes	No	No	No	No	No	No	No
Thakur, 2022 [42]	1	38	Yes	No	Yes	No	No	No	No	No	No	No	No
Thomson, 1989 [43]	0	59	Yes	No	No	Yes	No	No	No	No	No	No	No
Thomson, 1989 [43]	1	70	Yes	No	No	Yes	No	No	No	No	No	No	No
Trimble, 1983 [44]	0	53	Yes	No	No	Yes	No	No	No	No	No	No	No
Turton, 2022 [45]	1	18	Yes	No	Yes	No	No	No	No	No	No	No	No
Varghese, 2015 [46]	1	74	N/A	N/A	N/A	N/A	Yes	No	No	No	No	No	No
Xiao, 2017 [47]	0	83	Yes	No	Yes	No	No	No	No	No	No	No	No
Yang, 2016 [48]	1	52	Yes	No	No	Yes	No	No	No	No	No	No	No
Metzger, 1970 [49]	1	23	No	Yes	Yes	No	No	No	No	No	No	No	No
Alexander, 1973 [50]	1	21	No	Yes	Yes	No	No	No	No	No	No	No	No
Winters, 1955 [51]	1	19	Yes	No	No	No	No	No	No	No	No	No	No
Seymour, 1982 [52]	0	19	Yes	No	Yes	No	No	No	No	No	No	No	No
Goldschmidt, 2002 [53]	1	35	No	Yes	No	Yes	No	No	No	No	No	No	No
Enomoto, 2012 [54]	1	70	Yes	Yes	Yes	Yes	No	No	No	No	No	No	No
Vongsfak, 2020 [55]	0	65	Yes	No	No	Yes	No	No	No	No	No	No	No
Murakami, 1984 [58]	1	43	N/S	N/S	N/S	N/S	N/S	Yes	No	No	No	No	No
Hilbert, 1984 [59]	1	37	N/S	N/S	N/S	N/S	N/S	No	No	Yes	No	No	No
Noroy, 2020 [56]	1	68	Yes	No	No	Yes	Yes	No	No	No	No	No	No
Chue, 1975 [57]	0	27	No	Yes	Yes	No	No	No	No	No	No	No	No

N/A—not applicable; N/S—not specified.
